# Electro‐Active and Photo‐Active Vanadium Oxide Nanowire Thermo‐Hygroscopic Actuators for Kirigami Pop‐up

**DOI:** 10.1002/advs.202102064

**Published:** 2021-10-24

**Authors:** Rassoul Tabassian, Manmatha Mahato, Sanghee Nam, Van Hiep Nguyen, Araz Rajabi‐Abhari, Il‐Kwon Oh

**Affiliations:** ^1^ National Creative Research Initiative for Functionally Antagonistic Nano‐Engineering Department of Mechanical Engineering Korea Advanced Institute of Science and Technology (KAIST) 291 Daehak‐ro, Yuseong‐gu Daejeon 34141 Republic of Korea

**Keywords:** actuator, kirigami, photo‐active, thermo‐hygroscopic, vanadium oxide nanowires

## Abstract

Emerging technologies such as soft robotics, active biomedical devices, wearable electronics, haptic feedback systems, and healthcare systems require high‐fidelity soft actuators showing reliable responses under multi‐stimuli. In this study, the authors report an electro‐active and photo‐active soft actuator based on a vanadium oxide nanowire (VONW) hybrid film with greatly improved actuation performances. The VONWs directly grown on a cellulose fiber network increase the surface area up to 30‐fold and boost the hydrophilicity owing to the presence of oxygen‐rich functional groups in the nanowire surfaces. Taking advantage of the high surface area and hydrophilicity of VONWs, a soft thermo‐hygroscopic VONW actuator capable of being controlled by both light and electric sources shows greatly enhanced actuation deformation by almost 70% and increased actuation speed over 3 times during natural convection cooling. Most importantly, the proposed VONW actuator exhibits a remarkably improved blocking force of up to 200% compared with a bare paper actuator under light stimulation, allowing them to realize a complex kirigami pop‐up and to accomplish repeatable shape transformation from a 2D planar surface to a 3D configuration.

## Graphical abstract

An electro‐active and photo‐active soft actuator based on a vanadium oxide nanowire (VONW) hybrid film is developed with greatly improved actuation performances. The VONWs directly grown on a cellulose fiber network increase the surface area up to 30‐fold and boost the hydrophilicity owing to the presence of oxygen‐rich functional groups in the nanowire surfaces, resulting in superior thermo‐hygroscopic soft actuation.

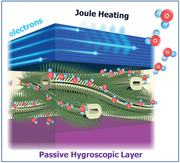

## 1 Introduction

Rapid advancements in material science and nanotechnology have led to the development of stimuli‐responsive active materials such as electroactive polymers,^[^ ^1^ ^]^ magneto/electrorheological fluids,^[^ ^2^ ^]^ shape memory alloys/polymers,^[^ ^3^ ^]^ liquid crystals,^[^ ^4^ ^]^ and so on. Inspired by the soft motion of animals and plants in nature, scientists have tried to take advantages of extraordinary properties of stimuli‐responsive materials to develop new soft robots that can not only mimic natural biological systems, but also tackle the shortcoming of existing noncompliant rigid mechanical systems. Various soft actuators have been proposed to fulfill the need for safer and more compliant systems such as pneumatic soft actuators,^[^ ^5^ ^]^ dielectric elastomer actuators,^[^ ^6^ ^]^ ionic actuators,^[^ ^7^ ^]^ photonic actuators,^[^ ^8^ ^]^ etc. The development of soft actuators has accelerated in recent years with the frequent emergence of new nanomaterials such as carbon nanotubes,^[^ ^9^ ^]^ graphene,^[^ ^10^ ^]^ covalent/metal organic frameworks,^[^ ^11^ ^]^ and MXene.^[^ ^12^ ^]^

Among several soft actuators, multi‐responsive thermo‐hygroscopic actuators have specific potential as they can be actively controlled both through electrical wiring and wirelessly.^[^ ^13^ ^]^ Most electro‐active soft actuators should have the electrical wires directly connected to the electrodes of the actuator. For example, in case of dielectric actuators or ionic actuators, the electric stimulation is transferred to the top and bottom electrodes of the actuator through external wiring. In addition, for pneumatic actuators, pressurized air is provided to a soft chamber through tiny tube inlets. However, since the main source of stimulus for hygroscopic actuators is thermal energy, the actuator can acquire the required heat remotely through light radiation.^[^ ^13b^ ^]^ Thermo‐hygroscopic actuators are mainly composed of two layers with different thermal and hygroscopic properties. The volume change in different materials occurs mostly due to thermal or hygroscopic expansion/shrinkage. However, these two effects work oppositely. It means by increasing the temperature, normal materials expand due to thermal expansion and shrink due to the moisture loss, which is called hygroscopic shrinkage. The superposition of these contradictory effects determines either if a material expands or shrinks by the temperature change. The volume change of most materials is dominated by thermal expansion as the hygroscopic effect is relatively negligible. However, recently some materials that the volume change is mostly dominated by hygroscopic effect have been introduced. It means, unlike normal materials, by increasing temperature the hygroscopic materials experience shrinkage due to the high degree of moisture loss that originated from their micro/nano‐structures. In thermo‐hygroscopic actuators, one passive layer is totally insensitive to moisture, which means that it is incapable of absorbing or desorbing moisture. Accordingly, this insensitive layer is called the passive hygroscopic layer. The other layer, which can adsorb and desorb considerable numbers of water molecules, is called the active hygroscopic layer. This layer is highly sensitive to moisture, such that its physical properties, like weight and volume, notably change when its moisture content changes. When such a bi‐layer structure is exposed to a heat source, the passive hygroscopic layer goes through thermal expansion while the active hygroscopic layer experiences a hygroscopic contraction due to evaporation of water molecules that were already accommodated inside this layer.^[^ ^14^ ^]^ This asymmetric volume change across the thickness leads to flexural bending of the actuator toward the contracted surface. The required heat for the actuation can be provided by radiation of electromagnetic waves like visible or infrared (IR) light. However, in specific conditions that electrical stimulation is preferred, a resistive electrode could be patterned on the actuator to provide the required thermal energy by Joule heating_._^[^ ^15^ ^]^ Such a patterned electrode can be used not only to realize electrical stimulation, but also to sense the real‐time deformation of the actuator.^[^ ^16^ ^]^ The extraordinary capability of thermo‐hygroscopic actuators to be stimulated by multiple sources of energy, along with their multi‐functionality (sensing and actuating), brings an exceptional opportunity to use this type of actuator in a variety of applications with different input requirements.

As discussed earlier, the most determining factor of a material to be used in this type of actuators is the hygroscopic property. Researchers have tried to synthesize varieties of new materials with high hygroscopic functionality to implement in thermo‐hygroscopic actuators. Owing to their high content of water, hydrogels are one type of hydrophilic cross‐linked structure that can go through considerable volumetric change by hygroscopic effect. Hydrogels usually have lower and upper critical solution temperatures, such that they repel their water content below and above that temperature. Tao Chen's group proposed a hydrogel actuator consisting of two active hygroscopic layers rather than one.^[^ ^17^ ^]^ In their proposed actuator, one layer is a hydrogel with lower critical solution temperature (LCST), while the other layer is another hydrogel with upper critical solution temperature (UCST). Switching the temperature between critical solution temperatures of the hydrogels repels water from one layer to the other, leading to contraction and swelling of the layers, respectively. While most research on hydrogel actuators has attempted to improve the hygroscopic properties of the proposed materials, some works have focused on improving the light absorbance, because the actuator needs to be stimulated by light.^[^ ^18^ ^]^ Although several hydrogels have been introduced for soft actuators until now, some major drawbacks remained unsolved, hampering the implementation of these materials in real field applications. Due to their high content of water, hydrogels are not generally stable in open air and can easily dehydrate. Furthermore, their reverse response is too slow and it may take a long time for them to recover to their original position, up to several hours.^[^ ^17^ ^]^ In some cases, hydrogels cannot even recover their original shapes without recharging them with bulk water, because they are not capable of attaining their lost moisture from humidity in open air.^[^ ^18^ ^]^

Another material that has been extensively used for thermo‐hygroscopic actuators is graphene oxide.^[^ ^13^, ^19^ ^]^ Graphene intrinsically has very high theoretical surface area, up to 2600 m^2^ g^−1^, because it consists of only one atomic layer of *sp* ^2^ carbon atoms.^[^ ^20^ ^]^ Owing to the presence of defects, graphene oxide has an even higher surface area, which is genuinely important regarding the absorbance and accommodation of water molecules. In addition, graphene oxide is superhydrophilic, capable of absorbing considerable amounts of water. On the edge and surface of the graphene oxide lattice, carbon atoms are bonded with a variety of oxygen‐rich functional groups that can readily interact with polar water molecules. Recently, MXene, one of other 2D materials, has been also used for thermo‐hygroscopic actuators.^[^ ^21^ ^]^ Although both graphene oxide and MXene have shown promising performance while used as the active material for thermo‐hygroscopic actuators, the synthesis route is so time‐consuming, difficult, and not cost‐efficient.

Metal oxide nanowires are other alternative that can be used for thermo‐hygroscopic actuators. Owing to their exceptional properties, metal oxide nanowires have been used in variety of applications such as energy storage,^[^ ^22^ ^]^ batteries,^[^ ^23^ ^]^ chemical sensors,^[^ ^24^ ^]^ and many others. Similar to 2D materials, metal oxide nanowires also have relatively high surface area due to their high aspect ratio of up to 1000.^[^ ^25^ ^]^ In addition, metal oxide nanowires contain large numbers of oxygen functional groups, making them highly interactive with water molecules in the air. However, in spite of their promising potential, metal oxide nanowires have so far not been utilized in any type of soft actuators.

**Scheme 1:**
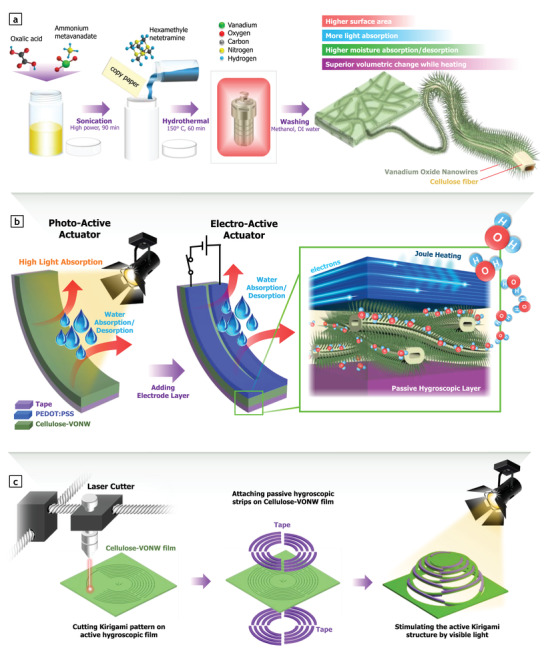
Experimental procedure of proposed cellulose‐VONW thermo‐hygroscopic actuator. a) Schematic illustration of synthetic route for growing VONWs on surface of cellulose fibers in copy paper. b) Actuation mechanism of proposed photo‐active and electro‐active thermo‐hygroscopic actuator containing hybrid cellulose‐VONW fibers stimulated by both electric potential and light. c) Fabrication of stimuli‐responsive kirigami pop‐up by cellulose‐VONW film for active shape transformation from 2D state to 3D configuration.

Herein, we synthesized vanadium oxide nanowires (VONWs) directly grown on cellulose fiber networks using hydrothermal process and realized electro‐thermal and photo‐active actuators for the first time (**Scheme** **1**a and Figure S1, Supporting Information). Seamlessly grown VONWs could remarkably enhance the surface area, water uptake, and light absorption in both the visible and infrared range, leading to improved hygroscopic volume change as well as intensified actuation performance. The proposed soft actuator could be stimulated by both light and electrical sources to be controlled wirelessly or through electrical wire connection (Scheme 1b). We also propose utilization of this newly developed actuator for the fabrication of a novel kirigami pop‐up to realize active shape transformation from a 2D planar state to a 3D configuration (Scheme 1c). The realized prototype suggests the high potential of metal oxide nanowire‐based actuators in a variety of future applications such as soft robotics, healthcare, and biomedical devices.

## 2 Characterization of Materials and Devices

**Figure 1:**
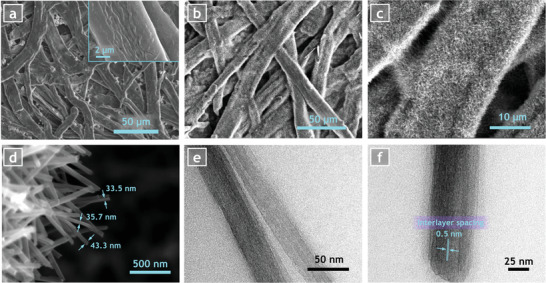
Microscopic characterization of morphology of samples. a) SEM image of the bare cellulose paper. The inset shows high magnitude SEM image of bare cellulose fibers. b) and c) SEM images of the VONWs grown on cellulose fibers with low and high magnifications. d) High resolution SEM image of VONWs grown on surface of cellulose fibers with diameters ranging from 30 to 50 nm. e) and f) TEM images of VONWs showing highly crystalline layered structure.

**Figure 2:**
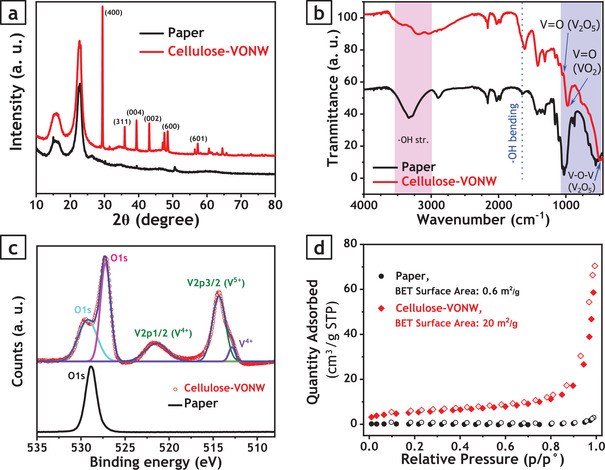
Chemical characterization of prepared bare paper versus hybrid cellulose‐VONW film. a) XRD data of samples showing distanced peaks at 29.5° (2*θ*) corresponding to (400) planes due to formation of VONWs. b) FTIR transmittance spectra of samples showing slight shift of peak for bending vibrations of —OH group from 1645 cm^−1^ for paper to 1616 cm^−1^ for cellulose‐VONW film. c) Comparison of XPS spectra of cellulose‐VONW film and bare paper in addition to corresponding deconvoluted peaks. d) Nitrogen adsorption–desorption isotherms of samples. Filled and empty symbols denote adsorption and desorption, respectively.

After conducting the hydrothermal process, samples were inspected by scanning electron microscopy (SEM) and transmission electron microscopy (TEM) to check the structure of the nanowires. While **Figure** **1**a shows the SEM images of bare cellulose paper, Figure 1b,c present the SEM images of VONWs grown on the cellulose fiber network. Present results show a uniform growth of nanowires over the whole surface of the paper. Higher magnification reveals that nanowires evenly covered the surface of every cellulose fiber, such that no voids or bare areas were observed, as shown in Figure 1c. More importantly, it can be clearly seen that VONWs are not randomly laid on the surface of the paper to make a flat film. Rather, they are grown on every cellulose fiber and perfectly follow the outer curvature of each fiber, suggesting the point that growth was initiated from the surface of the cellulose fibers and proceeded toward the outside (Figure 1d). The thickness of the VONWs grown by this method was found to vary between 30 to 50 nm, which range is very similar to what has been reported for hydrothermally grown VONWs in the literature.^[^ ^26^ ^]^ In Figure 1e,f, a different magnitude TEM images of VONWs is presented. As shown in the TEM images, the nanowires possessed a highly crystalline layered atomic structure. The interlayer spacing was found to be ≈0.5 nm. Growth of VONWs on the surface of paper, and its crystallinity, are further assessed by a series of spectroscopic analyses, with results presented in **Figure** **2**. High resolution X‐ray diffraction (XRD) patterns of the bare paper and cellulose‐VONW film are depicted in Figure 2a. The presence of intense XRD peaks of VONW, along with two broad peaks at 15.7° and 22.7° (2*θ*), confirmed the successful growth of the nanowires. The obtained XRD peak at 29.5° (2*θ*), corresponding to the (400) planes of VONW, as shown in Figure 2a, reaffirms the crystalline nature of the grown nanowires and supports the crystalline phases observed in the high‐resolution topography images (Figure 1e–f). Fourier transform infrared (FTIR) spectra, as shown in Figure 2b, reveal the functional groups present in both paper and cellulose‐VONW samples. The broad peaks observed in the wave number of 3600–3000 cm^−1^ for both samples correspond to stretching vibrations of the inter and intra molecular hydrogen‐bonded hydroxyl (—OH) groups. The FTIR transmittance peak at 1645 cm^−1^ for the bending vibrations of the —OH group, as observed for bare paper, is slightly shifted towards a lower wave number of 1616 cm^−1^ after successful growth of VONW on the surface. Most importantly, the obtained transmittance peaks at 1053, 978, and 503 cm^−1^ in the cellulose‐VONW sample confirm the presence of VONW on the paper surface, as seen in the characteristic stretching vibration of the V═O (V_2_O_5_), (VO_2_), and V—O—V (V_2_O_5_) functional groups, respectively. To understand the elemental configuration of paper and cellulose‐VONW, X‐ray photoelectron spectroscopic (XPS) analysis was performed; the obtained results are presented in Figure 2c. Revealing an XPS peak at 528.9 eV, the bare paper showed the presence of only one configuration of oxygen (O1s), whereas two distinct O1s XPS peaks corresponding to 529.3 eV for the oxygen from paper and 527.3 eV for the oxygen from VONW are observed in the case of cellulose‐VONW samples, confirming the growth of VONW on the surface of the cellulose fibers. The obtained dominant XPS peaks at 514.35 and 521.4 eV, corresponding to V2p_3/2_ and V2p_1/2_, respectively, for the cellulose‐VONW sample are related to the +5 oxidation state of vanadium pentoxide (V_2_O_5_). In addition, the presence of certain amounts of VO_2_ in the form of VONW is also observed in the XPS spectra of cellulose‐VONW, which shows an XPS peak at 512.95 eV.

It is obvious that the capacity for storing moisture increases with increase of the active surface area. Therefore, the active surface areas of the reported samples play significant roles in the actuation performances when samples are exposed to light sources. To analyze the effective surface areas of the bare paper and cellulose‐VONW, Brunauer–Emmett–Teller (BET) measurement was performed at liquid nitrogen temperature (77 K); the resultant isotherms are shown in Figure 2d. It can be observed that the presence of VONW on the surface of the paper increases the specific surface area from 0.6 (bare paper) to 20 m^2^ g^−1^ (cellulose‐VONW), indicating the higher order of surface adsorption–desorption phenomenon in the cellulose‐VONW sample.

**Figure 3:**
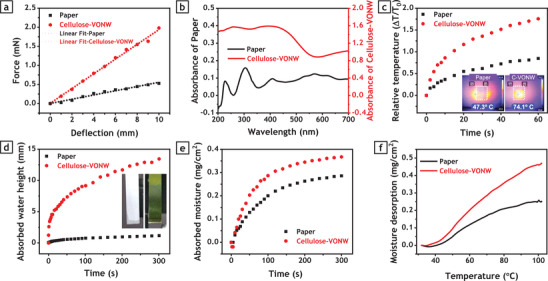
Physical characterization of bare paper versus hybrid cellulose‐VONW film. a) Force–deflection measurement data of samples for calculating bending stiffness. b) Light absorption spectra of samples showing superior absorption capability of cellulose‐VONW film in both visible and IR range. c) Temperature measurement of samples while exposed to light radiation with 10 cm distance from light source. The inset shows thermal images of bare paper and cellulose‐VONW film after exposure to the same light source. d) Water uptake of samples when tips of samples were immersed in water. The inset shows the real photograph of the samples after immersing the tip in water. e) Moisture absorption of samples in open air after being kept in oven (100 °C, 1 h) to remove most of stored moisture. f) TGA analysis of samples while varying temperature from 30 to 100 °C in 5 °C min^−1^ rate to show the moisture desorption capability of samples.

Afterward, the physical properties of the as‐prepared samples were assessed with multiple measurements. However, before further characterization, we found that commercial papers have an orthotropic nature. As illustrated in Figure S2, Supporting Information, a tensile test of samples, cut from commercial A4 paper, revealed that the tensile strength in the axial direction of the paper (along the length of the paper) is much higher than the tensile strength in the lateral direction (along the width of the paper). Calculated Young's modulus was found to be 422 kPa in the axial direction versus 166 kPa in lateral direction. Furthermore, ultimate elongation along the lateral direction was ≈4.5%, much higher than that of the axial direction (2%). The orthotropic properties of commercial paper originate from asymmetric orientation of the cellulose fibers, which occurs during the industrial fabrication process.^[^ ^27^ ^]^ To clarify the asymmetric orientation of cellulose fiber, SEM images were taken from the axial and lateral cross‐sections of the paper samples, as illustrated in Figures S2c and S2d, Supporting Information. As seen in the axial cross‐section SEM image, most of the cellulose fibers are perpendicular to the cross‐section plane and oriented toward this plane, such that their round cut cross‐section is observed. However, in the SEM image of the lateral cross‐section, plenty of cellulose fibers are observed that are oriented parallel to the cross‐sectional plane. This implies that the alignment of the fibers in the axial direction is dominant and causes higher tensile strength and lower extensibility in this direction. These considerations must be taken into account when choosing appropriate samples for the actuator, since the actuation performance relies highly on the expansion and contraction capability of the sample. Accordingly, all samples for this work were cut along the lateral direction of commercial paper, as paper showed higher elongation and lower tensile strength in this direction. To investigate the influence of VONW growth on the flexibility and bending capability of the samples, measurement of the bending stiffness was carried out as depicted in Figure S3, Supporting Information. Strips with dimensions of 7 mm ×  40 mm were cut from bare paper and VONW‐grown paper (cellulose‐VONW). One end of the samples was clamped while the other end was placed on a load cell (KYOWA LVS‐5GA), which was mounted on an adjustable moving stage. The positions of the free ends of the samples were measured using an accurate laser sensor. By moving the load cell upward, free ends of the samples were forced to move upward, which gradually bent the sample. The required force values for bending and tip displacement were recorded by the load cell and laser sensor, respectively. **Figure** **3**a shows the read‐out data from the as‐explained measurement system. As can be seen in the figure, both samples showed almost linear behavior with up to 10 mm of flexure. The calculated R‐square value for linear fitting was found to be 0.9952 in the case of the cellulose‐VONW sample and 0.9815 for bare paper. However, the cellulose‐VONW sample required much more force to bend compared to that needed for the bare paper, implying higher bending stiffness of the cellulose‐VONW sample. Accordingly, the bending stiffness of the investigated samples could be estimated by obtaining the slope of the linearly fitted lines in the force–deflection graph (Figure 3a). Using these calculations, the bending stiffness of the cellulose‐VONW sample was found to be 0.193 n m^−1^, ≈3.5 times of that of bare paper (0.055 n m^−1^). The increment of the bending stiffness was expected because a dense pile of VONWs uniformly grew on the surface of every cellulose fiber, leading to increased stiffness of the whole network.

Given that this type of actuator is expected to be stimulated by light, the capability of the prepared film to absorb light energy is critically important. Accordingly, the absorbance of the sample was measured within the visible and near infrared (NIR) range (wavelength: ≈200–2000 nm). As shown in Figure 3b, while the absorbance of bare paper is near zero, the cellulose‐VONW showed a much higher absorbance level in both visible and NIR range. As mentioned before, the growth of VONWs turns the color of the paper dark green, which is an intrinsically more light‐absorbing color compared to the white color of bare paper. However, the specific structure of the nanowires also greatly influences the absorbance. When light is illuminated onto a dense jumble of untidy nanowires, some portion of the photons are trapped inside and cannot escape such a complex surface, resulting in a higher level of absorbance.^[^ ^28^ ^]^ The ultimate objective of the as‐prepared film was to convert light energy to heat to induce thermal and hygroscopic expansion and contraction in the actuator. To evaluate this capability, samples with dimensions of 4 ×  5 cm^2^ were prepared and exposed to the same light source as would be used for the actuation test. The samples were placed at 10 cm distant from the light source while their temperature variation was monitored by a thermal camera, as shown in Figure S4, Supporting Information. As plotted in Figure 3c, with irradiation of samples with light, the temperature of the cellulose‐VONW sample ramped up quickly, while the temperature of bare paper rose sluggishly. This reaffirms the superior light absorption and higher photo‐thermal conversion efficiency of the cellulose‐VONW sample compared with bare paper. Interaction with water is another determining factor when considering an active material for hygroscopic actuators because the adsorption and desorption of moisture causes volumetric change. Growing VONWs on cellulose fibers makes them much more hydrophilic because of the intense presence of oxygen groups in the VONWs. As shown in Figure S5 and Video S1, Supporting Information, cellulose‐VONW showed a super‐hydrophilic characteristic such that when a droplet of water was placed on the sample, it was suddenly sucked into the film and did not remain on the surface. However, the bare cellulose paper steadily kept the water droplet on its surface. For better clarification of this effect, the water uptake was measured during the experiment. To do so, strips with dimensions of 6 mm  × 50 mm were cut from both samples and hung vertically. The bottom sides of the samples were simultaneously submerged in water while recording the process with an optical camera. The height of the absorbed water was extracted from the movie frames and matched to the corresponding time data points. The data obtained from the water uptake measurements are presented in Figure 3d. While the bare paper absorbed a negligible amount of water, the height of absorbed water for the cellulose‐VONW sample exceeded 13 mm. Moisture absorption ability of the proposed hybrid cellulose‐VONW film was also examined, as shown in Figure 3e. For this, samples were initially heated in the oven at 100 °C for 60 min to remove their moisture content. Afterward, the preheated samples were exposed to room temperature in open air while measuring their weight change. The obtained results revealed that the cellulose‐VONW film absorbed water molecules in air much faster and greater than did the bare paper. The speed of water absorption for the cellulose‐VONW sample was 68% higher than that of the bare paper sample within the first 50 s of exposure to the room environment. As shown in Figure 2, this occurs because VONWs mostly consist of V_2_O_5,_ with very rich oxygen content that can significantly interact with water molecules. Since the actuators are required to go through reversible actuation, both adsorption and desorption functionalities of moisture are profoundly important. Therefore, the moisture desorption rates of both samples were investigated by employing thermogravimetric analysis (TGA). The samples were heated from 30 to 100 °C under ambient condition with a heating rate of 5 °C min^−1^. The TGA machine recorded the weight loss of the samples during heating. The temperature of 100 °C was chosen as the upper limit to be sure that the recorded weight loss was due only to moisture desorption, rather than to the chemical decomposition of the samples. In this measurement, the hybrid cellulose‐VONW film showed a superior moisture desorption ability compared to bare paper. By heating the samples to 100 °C, the cellulose‐VONW film lost ≈470 µg moisture per square centimeter of film, while bare paper lost only 257 µg cm^−2^ at the same condition. Since VONWs interact with water molecules more actively, they adsorb more moisture at room temperature and accommodate higher numbers of water molecules on their surfaces. As a result, while heating the sample, cellulose‐VONW film has more content of moisture to release. All of the discussed properties greatly contribute to a high actuation performance and the VONWs remarkably improved the capability of the paper for use in thermo‐hygroscopic actuators in all aspects, as explained before.

## 3 Actuation Performance of Photo‐Active and Electro‐Active VONW Actuators

**Figure 4:**
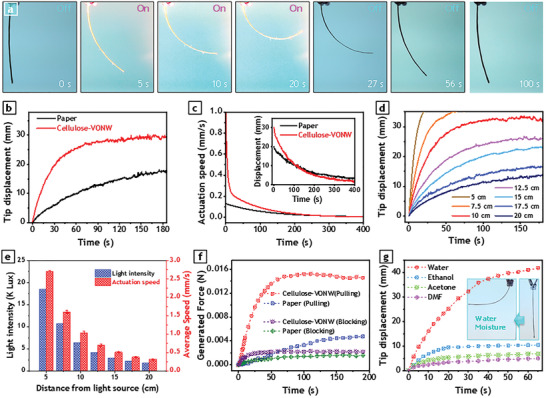
Actuation performance of thermo‐hygroscopic actuators in response to light stimulation. a) Optical photograph of cellulose‐VONW thermo‐hygroscopic actuator while subjected to light stimulation at 5 cm distance from light source. b) Comparison between the actuation response of cellulose‐VONW actuator and paper actuator when exposed to light stimulation at 10 cm distance. c) Actuation speed of proposed thermo‐hygroscopic actuators during reverse actuation when light stimulation is removed and actuators are cooling down. Inset shows tip displacement of actuators during cooling. d) Actuation response of cellulose‐VONW actuator when stimulated by the same light source at different distances. e) Intensity of light at different distances from light source (blue columns) and average speed of cellulose‐VONW actuator in first 10 s of actuation when exposed to light source at different distances (red columns). The average speeds were acquired from three samples and mean values and standard errors are plotted correspondingly. f) Measurement of blocking force (force generated in lateral direction) and pulling force (force generated in axial direction) of actuators in response to stimulation by light source at 10 cm distance. g) Actuation response of the cellulose‐VONW actuator when stimulated by vapors of various solvents. Inset shows the real photographs of the actuation when the actuator was exposed to water vapor.

Following the chemical and physical characterizations, the light‐stimulated actuators were fabricated for further analyses using a simple and straightforward fabrication route. For this purpose, a proper size of commercial Scotch packing tape was tightly attached to the proposed hybrid cellulose‐VONW film. The thickness of the attached tape was 80 µm. Then, pieces of as‐prepared actuators with dimensions of 6 mm ×  40 mm were cut and utilized for actuation measurement. The same procedure was used to make the actuators from bare paper. Fixed at the upper end, the actuators were placed vertically in front of a light source (Kolight DX400). The hygroscopic, active side of the actuators (VONW side) faced the light source to be maximally affected by the light. As presented in **Figure** **4**a, the proposed actuator under light exposure bent strongly toward the light and went back to its initial position after light stimulation was turned off. The tip displacement of the actuator was recorded using a highly accurate laser displacement sensor. The actuation displacements of both samples during light stimulation at a 10 cm distance are presented in Figure 4b. It has been shown that copy paper has a relatively high hygroscopic expansion coefficient of ≈0.1 C^–1^ and relatively low thermal expansion coefficient of (≈4–16) × 10–6 C^–1^ that makes its volume change to be dominated by the hygroscopic effect. It means cellulose paper shrinks while subjected to the temperature rise.^[^ ^15^ ^]^ Coating cellulose fibers by VONWs enhances the water storage capacity which further increases the hygroscopic expansion/shrinkage of the active layer. Although both cellulose and VONW experiences slight thermal expansion, their volume shrinkage due to moisture loss overwhelms the thermal expansion and causes the overall volume of the active layer to reduce by light stimulation. As can be seen in Figure 4b, the proposed cellulose‐VONW actuator showed much faster and larger actuation responses than those of the bare paper actuator and reaches a maximum displacement of ≈30 mm. This level of actuation is very intriguing, compared to that of other thermo‐hygroscopic actuators.^[^ ^29^ ^]^ The notable improvement of the actuation performance originates from the superior hydrophilic capability of VONWs to interact with water molecules. As discussed earlier, the VONWs are capable of absorbing and desorbing more moisture, boosting the hygroscopic volumetric expansion and contraction. This effect not only improves the degree of actuation, but also profoundly increases the actuation speed. In addition, the proposed cellulose‐VONW possesses a 30 times higher surface area, which boosts the cooling rate and makes the actuator to reabsorb its lost moisture quickly. A high actuation output makes it possible to get any level of stable displacement within the maximum limit of the actuator. As shown in Figure S6, Supporting Information, a constant stimulation could be applied for a certain amount of time until the actuator reaches the desirable displacement. Afterward, high frequency stimulation could be applied to keep the displacement constant. Indeed, the high frequency stimulation causes the actuator to oscillate around the desirable displacement level (Figure S6b, Supporting Information). However, it is possible to minimize deviation of the response from target displacement by choosing appropriate amplitude and frequency of the stimulation and a feedback control strategy can be practically applied to realize more accurate control systems.

The reverse actuation, when the source of stimulation is removed, is of great importance for all types of thermo‐responsive actuators. As the reverse actuation of thermo‐responsive actuators is governed by the cooling rate of the samples and cannot be actively boosted, these types of actuators usually show sluggish dynamic behavior during reverse actuation. Accordingly, the actuation speed of the proposed actuator during cooling state was investigated by stimulating samples for the same duration and leaving them to naturally cool down while measuring their displacement (Figure 4c). For more details on the speed calculations, please check Figure S7, Supporting Information. As can be observed in Figure 4c, the proposed hybrid cellulose‐VONW actuator showed a much higher absolute speed during reverse actuation than did the bare paper actuator. The higher actuation speed can also be inferred from the displacement diagram, in the inset of Figure 4c. Since the speed is defined as the first time‐derivative of the displacement, the slope of the displacement graph indicates the speed of actuation. As shown in the inset of Figure 4c, the displacement curve of the cellulose‐VONW actuator has a much sharper slope toward the zero axis than does the bare paper actuator, reaffirming the faster actuation of the proposed actuator. Similar to the conventional fins in heat exchanger systems, here also VONWs improve the cooling rate of the sample. The higher surface area of VONWs provides a larger accessible surface to exchange heat between cellulose fibers and air molecules, resulting in a faster cooling process. As a result, the reverse actuation occurs much faster and actuator recovers its shape more quickly.

The influence of light intensity on the actuation performance of the proposed cellulose‐VONW actuator was investigated by varying the distance of the actuator from the light source. The distance from the light source was changed from 5 to 20 cm, at 2.5 cm intervals, and the tip displacement was recorded using a laser sensor, with results plotted in Figure 4d. As the sample approached the light source, the output displacement of the actuator quickly ramped up. In the cases of 5 and 7.5 cm distance, the actuation was so prompt that the tip displacement of the actuator quickly moved out of the measurement limit of the laser sensor, over 36 mm. As illustrated in Figure 4e, changing the distance from the light source alters the light intensity at the actuator position. Theoretically, the light intensity is inversely proportional to the square of distance, indicating that it increases dramatically by approaching the light source. As a result, the actuator can absorb more energy and facilitate evaporation of the pre‐stored moisture at an elevated rate. As shown in Figure 4e, the escalation of the light intensity increased proportionally with the actuation speed (a comparison of the actuation speed with other hygroscopic actuators is provided in Table S3, Supporting Information).

In addition to the constant stimulation, the cellulose‐VONW actuator was also tested for cyclic stimulation. As displayed in Figure S8, Supporting Information, the proposed actuator showed very uniform response to cyclic stimulation. Cyclic stability of the actuator was also measured by applying the stimulation for 170 cycles. As could be seen in Figure S9, Supporting Information, normalized peak‐to‐peak displacement of the actuator remained almost constant during the test and is barely deviated from one.

The main objective of all types of experimental actuators is ultimately their use in real applications. Therefore, the mechanical output of the actuator, the generated force, is crucially important when considering the potential of an actuator for implementation in a real mechanical system. Accordingly, the force generation capability of the proposed actuator was investigated in two directions, lateral and axial.^[^ ^15^ ^]^ During the operation time, the actuators were stimulated by the light source and their generated forces were measured with a load sensor (KYOWA LVS‐5GA). The generated force in the lateral direction is normally called the blocking force, and is very commonly measured for various types of actuators. The measurement of the blocking force revealed that the force output of the proposed actuator follows a profile similar to that of the displacement output during the stimulation time span. As shown in Figure 4f, by applying stimulation, the generated blocking force increased to a certain level and then remained relatively constant, similar to that observed for tip displacement of the actuator. The light stimulation causes evaporation of moisture in the hygroscopic active layer until equilibrium between the temperature of the layer and the moisture content is achieved, after which no further evaporation occurs. The moisture evaporation leads to the tendency of the actuator to bend toward the hygroscopic active layer. However, since the free end of the actuator is blocked by the load cell and the deformation is constrained to some extent, the vacancy induced by moisture evaporation generated a negative stress in the layer. As expected, the proposed cellulose‐VONW actuator showed a notably higher blocking force than that of the bare paper actuator under the same stimulus conditions. Furthermore, the cellulose‐VONW actuator reached its steady condition much faster than did the bare paper actuator. Since the cellulose‐VONW absorbs the light energy in a higher rate, as discussed before in Figure 3c, it could reach its equilibrium state of moisture and temperature much faster than bare paper, leading to faster stabilization of the generated blocking force. The superior capability of the proposed cellulose‐VONW actuator was more distinctly obvious when the axial force generated due to light stimulation was measured. When the actuator bent due to external stimulation, the distance between the two ends of the actuator tended to decrease owing to the induced curvature. However, if this contraction in the axial direction is constrained by the load cell, a pulling force is generated in this direction. Pulling force measurements revealed a much more distinct difference between the proposed and control samples. As shown in Figure 4f, owing to outperforming hygroscopic properties, the VONWs enhanced the pulling force of the actuator by almost 200%. Another intriguing point that can be deduced from the generated force measurements in this type of actuator is their superior capability in generating pulling force rather than blocking force. A comparison of Figure 4f reveals that the cellulose‐VONW actuator generates a more than fivefold higher pulling force than blocking force. Even the generated pulling force of the bare paper actuator is 3 times higher than its blocking force in the same stimulation conditions. These results suggest that, to extract a maximum force‐generating capability of an actuator, it is better to implement hygroscopic actuators in layouts that are able to utilize the axial force rather than the lateral force.

Since the moisture is the main factor providing the driving force of the actuation, the response of the proposed cellulose‐VONW actuator was also measured when it was exposed to the vapor of various solvents. To produce adequate vapor for the stimulation, a mini humidifier (260 mL, 5 V) containing target solvents was placed below the actuator as shown in Figure S10, Supporting Information. The tip displacement of the actuator was measured since the humidifier turned on. Water, acetone, ethanol, and dimethylformamide (DMF) were tested as stimulating solvents and the corresponding response data was potted in Figure 4g. The response of the actuator to water moisture is far better than that of other solvents. The superior effectiveness of water as a stimulating solvent could be ascribed to the highest polarity of water molecules among all tested solvents. However, the other factor is the molecular size of the sample. For example, in spite of similar relative polarities of the acetone (0.355) and DMF (0.386) molecules, the response of cellulose‐VONW actuator under the exposure of acetone vapor was greater than the response due to DMF. Indeed, the size of DMF molecules is much larger than that of acetone, which makes it harder for DMF molecules to intercalate between the VONWs. Another interesting point about this experiment was the opposite direction of actuation with respect to light stimulation. Unlike light stimulation, in case of moisture stimulation, the actuator bent toward the passive layer since the surge of moisture into the active layer (cellulose‐VONW) caused this layer to expand and bend the actuator toward the opposite direction.

**Figure 5:**
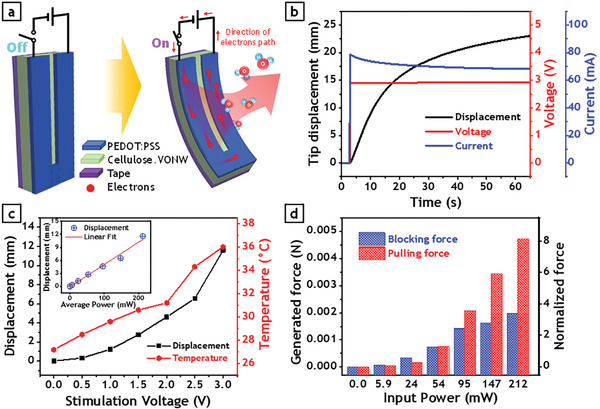
Actuation performance of proposed thermo‐hygroscopic cellulose‐VONW actuators in response to electrical stimulation. a) Schematic layout and actuation mechanism of electrically stimulated thermo‐hygroscopic actuator. b) Input voltage, induced current in actuator electrode, and transient displacement response of cellulose‐VONW actuators when subjected to electrical stimulation. c) Maximum displacement and temperature of the actuator while stimulated by different input voltages. Inset shows linear correlation between corresponding input power and output displacement. d) Measurement of generated blocking force and pulling force by electrically stimulated cellulose‐VONW actuators in response to different input powers (left vertical axis shows the absolute values of the generated force while right vertical axis shows the non‐dimensional values of the generated force normalized with respect to the weight of the actuator).

After confirming the outperformance of the cellulose‐VONW sample, this actuator was further investigated for another type of stimulation. Applying a conductive coating, the proposed cellulose‐VONW actuator was used as an electroactive actuator. A U‐shape conductive electrode was patterned on the surface of the as‐prepared actuators to provide a path for the movement of electrons on top of the hygroscopic active layer, as depicted schematically in **Figure** **5**a. Briefly, electrode solution was prepared by adding 5 v% dimethyl sulfoxide (DMSO) to poly‐ethylene dioxythiophene:poly‐styrene sulfonate (PEDOT:PSS) solution. The conducting polymer was drop‐casted on the active surface of the actuators and dried on a hot plate (70 °C) for 30 min. After drying the electrode solution, using a laser cutter, a groove was engraved on the surface of the PEDOT:PSS layer to make the U‐shape pattern (Figure S11, Supporting Information). The engraved groove is required to be adequately deep to prevent a shortcut between the two branches of the U‐shape pattern. More details about the fabrication process can be found in the Experimental section. The sheet resistance of the as‐prepared PEDOT:PSS conductive path was 1.2 Ω/square. By applying an electric potential to the end points of the U‐shape electrode, a flow of electrons is induced through the U pattern, resulting in Joule heating. The generated heat is absorbed by the actuator and used as stimulation to trigger moisture evaporation inside the hygroscopic active layer, as well as thermal expansion of the non‐hygroscopic layer. The mechanical response of the as‐prepared actuator to electric stimulation was investigated, with results presented in Figure 5b. A relatively low voltage of 3V was applied to the actuator and the tip displacement was recorded by the laser sensor. As can be observed in Figure 5b, by applying electric stimulation, the actuator bent due to the heat generated in the electrode, similar to the way it bent due to light stimulation. The speed of actuation was initially fast and gradually degraded as the actuator approached its steady state. The current passing through the U‐shape electrode during actuation can also be measured using an extra resistor in series with the actuator (Figure S12, Supporting Information). By applying the voltage, the electric current in the circuit jumped to a maximum value and then decreased gradually during actuation. The variation of electric current is due to the variation of the resistance of the actuator electrode during actuation.^[^ ^16^ ^]^ Using the measured current, the electrical power consumed by the actuator can be calculated, as explained in the Supporting Information. Figure 5c shows the temperature change and corresponding displacement during 10 s of actuation while applying different levels of input electrical potential. It can be inferred from these results that, by elevating the input potential, the generated heat in the electrode rises up leading to the increase of actuator temperature and consequently output displacement. Although the increment of displacement is of second order with respect to the input voltage, it increases linearly with the input power as displayed in the inset of Figure 5c. The linear fitted line to the measured data of this graph yielded the R‐square of 0.985.

Force generated by the actuator using electrical stimulation was also investigated. Figure 5d displays the measured blocking force and pulling force at different input powers. It can be seen that, by increasing the input power, the force generated by the actuator also increases sharply. However, the increment of the pulling force is more notable compared to that of the blocking force. As could be seen in this graph, the proposed cellulose‐VONW actuator was able to generate a pulling force over 8 times of its weight when stimulated by only 3 V input corresponding to 212 mW electrical power.

Although patterning electrode on the actuator facilitates electrical stimulation, it can also affect the actuation mechanism in some ways. First of all, PEDOT:PSS is a hydrophilic material owing to its sulfonated groups. It means the electrode itself can also absorb/desorb water and actively contribute to the actuation.^[^ ^14^ ^]^ However, at the same time it covers the surface of cellulose‐VONW film, resulting in partially hindering the evaporation and trapping the water inside the film. Although, there are still some nano‐channels in PEDOT:PSS that allow for water exchange between the cellulose‐VONW film and the surrounding environment, the evaporation rate is expected to be much lower than the film without conductive polymer coating. The superposition of these two antagonistic effects can determine how PEDOT:PSS coating influences the output displacement of the actuator. To better find out these effects the moisture absorption of the cellulose‐VONW actuators with and without PEDOT:PSS coating was measured using the same method as discussed before. As presented in Figure S13a, Supporting Information, the actuator with PEDOT:PSS coating showed much slower rate of moisture absorption at the room temperature implying the sluggish water exchange with the surrounding environment. The actuators were also stimulated by the light source at 10 cm distance to check how PEDOT:PSS coating affects their performance. As shown in Figure S13b, Supporting Information, the actuator with PEDOT:PSS electrodes showed a lower level of displacement compared to the one without electrode. It means that decreasing the evaporation rate of cellulose‐VONW film has more overwhelming influence on actuation than hygroscopic properties of the PEDOT:PSS.

All of the discussed measurements confirm the important role of VONWs in the improvement of thermo‐hygroscopic actuation and suggest the remarkable potential of this type of actuator for implementation in practical engineering applications.

## 4 Demonstration of Kirigami Pop‐Up

**Figure 6:**
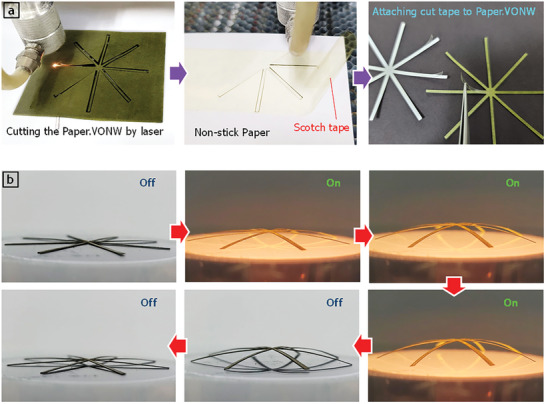
Demonstration of spider‐shape kirigami pop‐up utilizing proposed thermo‐hygroscopic cellulose‐VONW actuators. a) Fabrication steps for making spider‐shape kirigami pop‐up using a laser cutter. b) Optical photographs of proposed spider‐shape pop‐up during light stimulation and recovery.

Using the cellulose‐VONW actuator, two active kirigami pop‐up structures were designed to further demonstrate the capability of the proposed actuator. Kirigami is a subclass of origami that includes cutting the paper rather than only folding. Origami and kirigami are ancient techniques used for transformation of 2D papers into 3D structures without use of glue or other types of attachments.^[^ ^30^ ^]^ Although most of the recent applications of kirigami are still limited to passive implementation of these structures, thanks to the development of new soft actuators, a new field is emerging that involves incorporating the actuators in kirigami structures to actively control the structural transformation. In this work, two kirigami structures are presented utilizing the proposed cellulose‐VONW actuator, capable of being stimulated by light to pop up from the surface and transform from a 2D flat sheet to a 3D structure. In the first design, a spider‐shape pattern with eight legs was made with the cellulose‐VONW film using the laser cutter. Each leg has 32 mm length and 1.5 mm width. Then, the Scotch tape strips with the same size were attached on the spider‐shape cellulose‐VONW film as the passive layer. The fabrication steps for this design are shown in **Figure** **6**a. After preparation of the pop‐up actuator, it was stimulated by the same light source. As illustrated in Figure 6b and Video S2, Supporting Information, by applying the light stimulation, the legs started curving downside. Since the pattern is symmetric, the free ends of the legs pushed against the floor and lifted the center of the spider upward. By turning off the light stimulation, the legs were fully recovered to their initial shapes and the pop‐up structure returned back to flat configuration.

**Figure 7:**
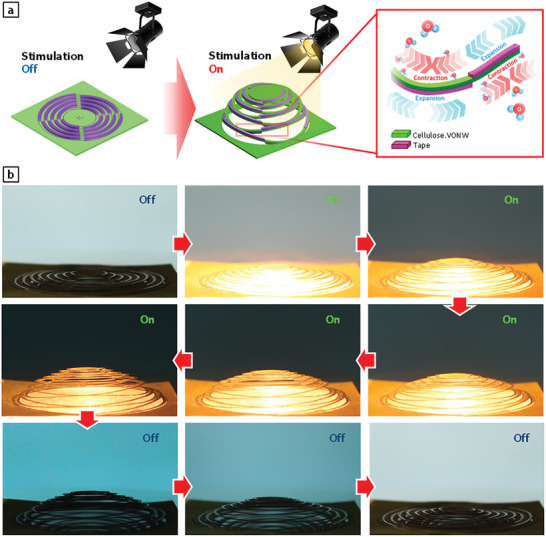
Demonstration of concentric kirigami pop‐up employing thermo‐hygroscopic cellulose‐VONW actuators. a) Schematic illustration of working mechanism and shape transformation from 2D planar state to 3D configuration. b) Optical photographs of proposed active kirigami during light stimulation and recovery.

The second kirigami pop‐up possessed a more complex pattern. This specific pattern contained multiple pairs of arcs with different radiuses (30 to 6 mm) centering at the same point. Cellulose‐VONW film with a size of 70 mm × 70 mm was prepared and the suggested pattern was cut on it using the laser cutter (Figure S14, Supporting Information). As schematically depicted in Figure 6a, the proposed kirigami was expected to transform from state A (2D) to state B (3D) when exposed to light stimulation. A closer look at the expected transformed structure reveals that every arc strip in the structure requires a partially upward curvature and partially downward curvature at the same time. This means one portion of each strip needs to actuate upward while the other portion actuates downward. To realize such a multi‐curvature actuation, passive hygroscopic layers (Scotch tape) must be attached on opposite sides of the hybrid cellulose‐VONW film sequentially (For more details, please check the Experimental Section). As illustrated in **Figure** **7** and Video S3, Supporting Information, by applying light stimulation, the well‐designed kirigami started to pop up from the surface and transform into a 3D structure. In this design, all the links work as independent multi‐curvature actuators, which are unified into an integrated kirigami structure. By applying light stimulation, parts of the links that had tape attached on the top side bent downward, while portions that had tape attached on the bottom side bent upward due to the expansion of the tape and evaporation of moisture from the cellulose‐VONW film. Actuation of all links together realized transformation of the whole structure from a 2D flat sheet to a pop‐up 3D structure. The proposed smart kirigami also realized a fully reversible transformation such that, by removing the light source, it went back to its 2D configuration and fully recovered the initial shape. Such outstanding photo‐active shape transformation has promising potential for implementation in future technologies such as soft robotics and MEMS with remote control strategy.

## 5 Conclusion

In this study, we report electro‐active and photo‐active thermo‐hygroscopic actuators based on vanadium oxide nanowires grown on cellulose papers. Using a cost‐effective hydrothermal process, the vanadium pentoxide nanowires (VONWs) were uniformly grown on a commercial copy paper having cellulose fiber networks. The seamlessly grown VONW nanowires greatly increased the active surface area up to 3000% and notably enhanced the hydrophilicity of the surface, capable of boosting interaction with polar H_2_O molecules due to the presence of a high content of oxygen functional groups in VONWs. The increased surface area along with the excellent hydrophilicity of the proposed hybrid cellulose‐VONW film led to much higher water absorption and desorption in open air compared with bare cellulose paper. In addition to the water uptake, the unique structure of VONWs influenced the interaction of the surface with projected light. It is critically important to point out that thermo‐hygroscopic actuators are mostly stimulated by light sources and the light‐absorption ability can profoundly affect the actuation mechanism. Careful investigation of the samples revealed that VONWs intensively improved the light absorption of the cellulose paper (more than tenfold) in both visible and IR wavelengths. The improved light absorption could be due to the intrinsic dark color of the VONWs, as well as their clumsy structures, which somehow trap light and allow the actuator to be remotely controlled. These exceptional properties of the proposed hybrid cellulose‐VONW film were employed to make a high‐performance thermo‐hygroscopic actuator simply by attaching cheap and ubiquitous packing tape as a passive hygroscopic layer. The as‐prepared actuator showed deformation and a force superior to that of a bare paper actuator when exposed to the same light source. Most conventional thermo‐hygroscopic actuators show sluggish reverse actuation due to a lack of swift moisture desorption and slow cooling rate. However, the novel cellulose‐VONW actuator shows prominent performance in reverse actuation, such that the average actuation speed for the proposed actuator was 3 times higher than that of the bare paper actuator. Most importantly, the high moisture absorption and desorption rate of the cellulose‐VONW film led to a 3 times higher output force, which is significant considering the real implementation in practical application fields. The present study demonstrated that the proposed thermo‐hygroscopic actuator can be directly applied to a complex kirigami pop‐up to accomplish active shape transformation from a 2D planar surface to a 3D configuration using remotely controlled light sources. The intriguing findings of the present study suggest potential applications of metal oxide nanowires in not only thermo‐hygroscopic actuators, but also photo‐active actuators.

## 6 Experimental Section

### Chemicals

Oxalic acid dihydrate and ammonium metavanadate were bought from Daejung Chemical, Korea. Hexamethylenetetramine and dimethyl sulfoxide were purchased from Sigma Aldrich, Germany. Ethanol 99.5% was attained from Samchun Chemical, Korea. PEDOT:PSS solution (Clevios PH 1000) was purchased from Heraeus, Germany. The copy paper for growing VONWs obtained from Double A Co., Thailand.

### Growth of VONW on Copy Paper

1.2 g of ammonium metavanadate and 2.32 g of oxalic acid dihydrate were mixed in 50 mL deionized (DI) water and sonicated for 90 min. After sonication, the solution was kept at room temperature to cool. Then, 0.176 g of hexamethylenetetramine was added to the solution and stirred with a glass rod to fully dissolve. The as‐prepared solution was transferred to a 100 mL Teflon reaction chamber. Then, a copy paper with size of 50 mm × 70 mm was soaked in the solution. The Teflon chamber was then put into a tightly closed autoclave for hydrothermal treatment. The autoclave was transferred to an oven, which had already been heated to 150 °C, and kept there for 60 min. When the hydrothermal process completed, the autoclave was taken out of the oven and kept at room temperature to cool. Later, the VONW‐grown sample was collected from the autoclave and carefully washed with DI water and ethanol. Finally, the as‐prepared sample was dried in oven to remove extra water and ethanol.

### Material Characterization

SEM images were taken using a field emission SEM‐FEI Magellan400. TEM images were taken by a 300 kV Tecnai G2 F30 S‐Twin, made by FEI Company. XRD data were obtained using a Thin‐Film X‐Ray Diffractometer (Ultima IV) made by the RIGAKU Company. FTIR data were obtained by FTIR Spectrometer (Nicolet iS50), a product of Thermo Fisher Scientific Instrument. XPS analysis was carried out using an XPS (K‐alpha) made by Thermo VG Scientific. BET data were obtained from an ASAP 2020, made by Micromeritics Instrument Corporation, USA. The light absorption measurement was conducted utilizing a UV–VIS/NIR Spectrophotometer‐Lambda 1050 made by Perkin Elmer.

### Actuator Fabrication

Scotch packing tape was gently attached to the surface of both bare paper and cellulose‐VONW film, without any trapped bubbles or wrinkles. 6 mm × 40 mm pieces of the sample were cut and used as light‐responsive actuators. To allow stimulation of the actuators by the electrical signal, an electrode layer was also cast on the as‐prepared samples. First, 100 µL of DMSO was added to 2 mL of % PEDOT:PSS solution and stirred with a magnet stirrer at a rotational speed of 300 rpm for 2 h. Adding DMSO to the PEDOT:PSS increased the electrical conductivity of the electrode. After this, prepared samples were placed on a glass plate in such a way that the active hygroscopic side was facing upward. All edges of samples were then constrained using Kapton tape. 0.5 mL of the prepared PEDOT:PSS solution was drop casted on the samples and dried on a hot plate (70 °C, 30 min). Later, straight lines with lengths of 35 mm were engraved by a laser cutter on the dried PEDOT:PSS electrode, starting from one end and with the same distance from the long edges. In this step, the power of the laser cutter needed to be carefully adjusted to fully engrave the electrode without hurting the other layers. Two narrow strips of copper foil were attached to the both ends of the U‐shape PEDOT:PSS electrode using silver paste. Eventually, the wiring connection part was encapsulated between two layers of adhesive tape to protect the connections from detachment.

### Actuation Measurement

To investigate the actuation response to light stimulation, the prepared actuators were gripped at one end and hung vertically. A photography lightening device (Kolight DX400) was placed at specific distances ranging from 5 to 20 cm as a light source. The light was radiated to the active hygroscopic surface of the actuators, while the tip displacement of the free end of the actuators was simultaneously recorded by a very accurate laser sensor (Keyence, LK031). The response to electric stimulation was also measured in a similar manner. However, for this measurement, the actuator with U‐shape PEDOT:PSS electrode was mounted on the gripper. The connected copper strips were supplied by an input potential from a Universal Power Module made by Quanser. Both the input voltage and output signal of the laser displacement sensor were simultaneously recorded using a National Instrument DAQ board (BNC‐2120) connected to an NI PXI‐6070E chassis. The force generated by the actuators in response to both light and electric voltage was measured using a 50 mn load cell (LVS‐5GA) made by KYOWA, Japan.

### Fabrication of Active Kirigami Pop‐Up

First, a 70 mm × 70 mm piece of copy paper was coated with VONWs. Then, a kirigami pattern was accurately cut on the sample using a laser cutter. The proposed kirigami pattern contained multiple concentric arc pairs with a singular center. Each pair consists of two 170° arcs facing each other and sharing the same center. From one pair to the next, the radius of the arcs decreased by 1.5 mm and the orientation of the pairs changed by 90°. For example, if the arcs in the first pair (with a radius of 30 mm) were facing up‐down, the arcs in the next pair (with 1.5 mm reduced radius) were facing right‐left. This sequence was repeated many times while varying the radius from 30 to 6 mm. Afterward, a 6 cm wide piece of Scotch tape was attached on the non‐stick paper. A pattern was cut on the Scotch tape similar to the proposed kirigami. However, the pattern contained concentric circles rather than 170° arcs (same radius as kirigami pattern). Furthermore, two perpendicular lines crossed at the center of the circles, dividing them into four equal pieces. Having cut such a pattern, quarter‐circle strips of Scotch tape with 1.5 mm width were obtained such that for each circle strip on the kirigami paper there were four quarter‐circle tape strips to be attached. From each set of quarter‐circle tape strips, two quarter‐circle strips were attached to the top of the corresponding circle strip of the kirigami paper and two were attached to the bottom, without overlapping. This means that each circle strip of kirigami paper was divided into four regions and the attachment side sequentially switched between top and bottom. The position of the tape strips on the kirigami paper needed to match with the expected transformed shape of the kirigami. When the proposed kirigami was transformed from 2D state to the 3D configuration, each circle strip experienced positive curvature in two regions and negative curvature in the other two regions. Since the tape strips work as passive hygroscopic layers, the heat stimulation induced bending toward the opposite side of the tape attachment. Therefore, the position of the tape defined the positions of curvatures and needed to match with the expected curvatures in the transformed configuration.

## Author Contributions

R.T. and M.M. contributed equally to this work. The manuscript was written through contributions of all authors. All authors have given approval to the final version of the manuscript.

## Conflict of Interest

The authors declare no conflict of interest.

## Supporting information

Supporting Information

Supplemental Video 1

Supplemental Video 2

Supplemental Video 3

## Data Availability

The data that support the findings of this study are available from the corresponding author upon reasonable request.
