# Supplementary material for: Electro‐Active and Photo‐Active Vanadium Oxide Nanowire Thermo‐Hygroscopic Actuators for Kirigami Pop‐up
Source: Adv Sci (Weinh). 2021 Oct 24;8(23):2102064. doi: 10.1002/advs.202102064 (PMC8655174; doi:10.1002/advs.202102064)
Supplement: Supplementary file 1 — Supporting Information [file ADVS-8-2102064-s003.pdf]

© 2021 Wiley-VCH GmbH

ADVANCED SCIENCE
Open Access

Supporting Information

for *Adv. Sci.*, DOI: 10.1002/advs.202102064

Electro-Active and Photo-Active Vanadium Oxide Nanowire Thermo-Hygroscopic Actuators for Kirigami Pop-up

Rassoul Tabassian[‡], *Manmatha Mahato*[‡], *Sanghee Nam, Van Hiep Nguyen, Araz Rajabi Abhari, Il-Kwon Oh*[*]

Supporting Information

Electro-Active and Photo-Active Vanadium Oxide Nanowire Thermo-Hygroscopic Actuators for Kirigami Pop-up

Rassoul Tabassian[‡], Manmatha Mahato[‡], Sanghee Nam, Van Hiep Nguyen, Araz Rajabi Abhari, Il-Kwon Oh[*]

Dr. R. Tabassian, Dr. Manmatha Mahato, S. Nam, V. H. Nguyen, Dr. A.R. Abhari, Prof. I.-K. Oh.
National Creative Research Initiative for Functionally Antagonistic Nano-Engineering, Department of Mechanical Engineering, Korea Advanced Institute of Science and Technology (KAIST), 291 Daehak-ro, Yuseong-gu, Daejeon 34141, Republic of Korea

[*] Correspondence and requests for materials should be addressed to I.-K. Oh (Email: ikoh@kaist.ac.kr)

Keywords: Vanadium Oxide Nanowire; Thermo-Hygroscopic; Actuator; Kirigami; Photo-active; Electro-active

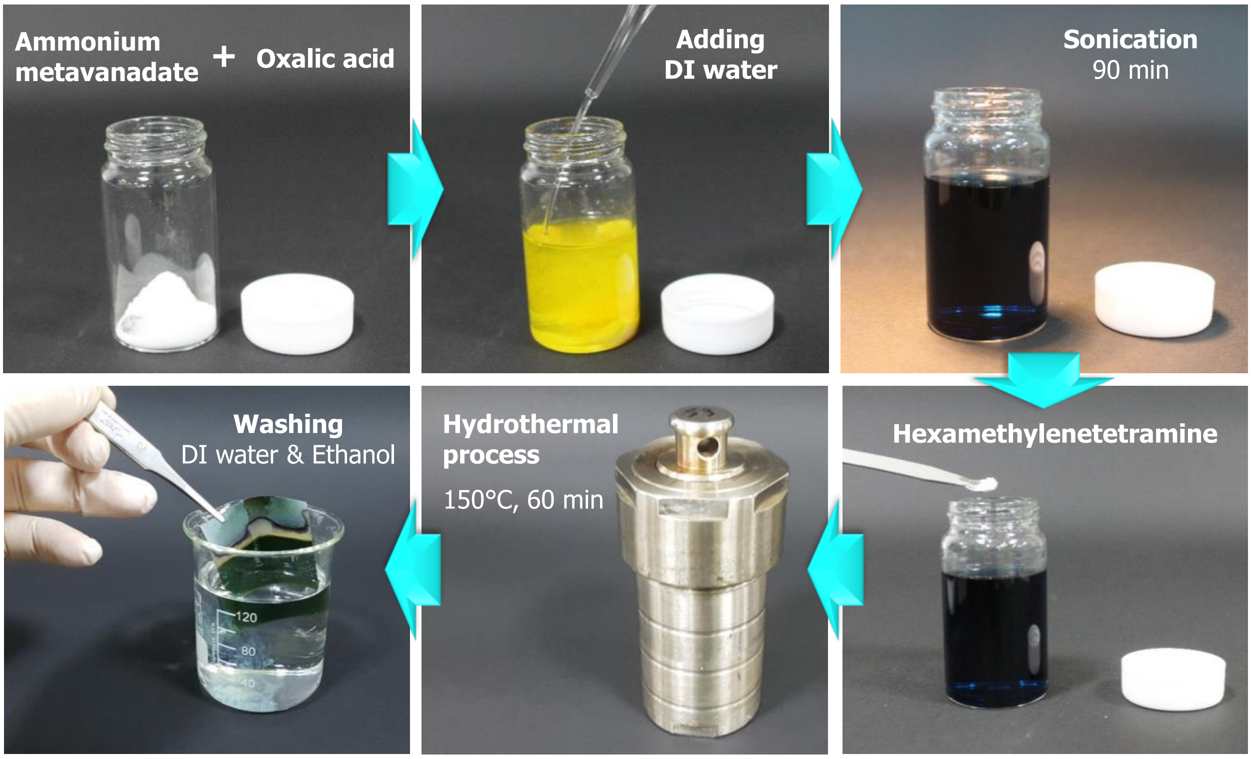

Figure S1. Synthesis steps for growing VONWs on surface of copy paper: mixing ammonium metavanadate and oxalic acid in water, sonicating for 90 min, adding hexamethylenetetramine, submerging piece of paper in solution, and putting paper in autoclave for hydrothermal process (150 °C, 60 min). Cooling and washing samples with DI water and ethanol.

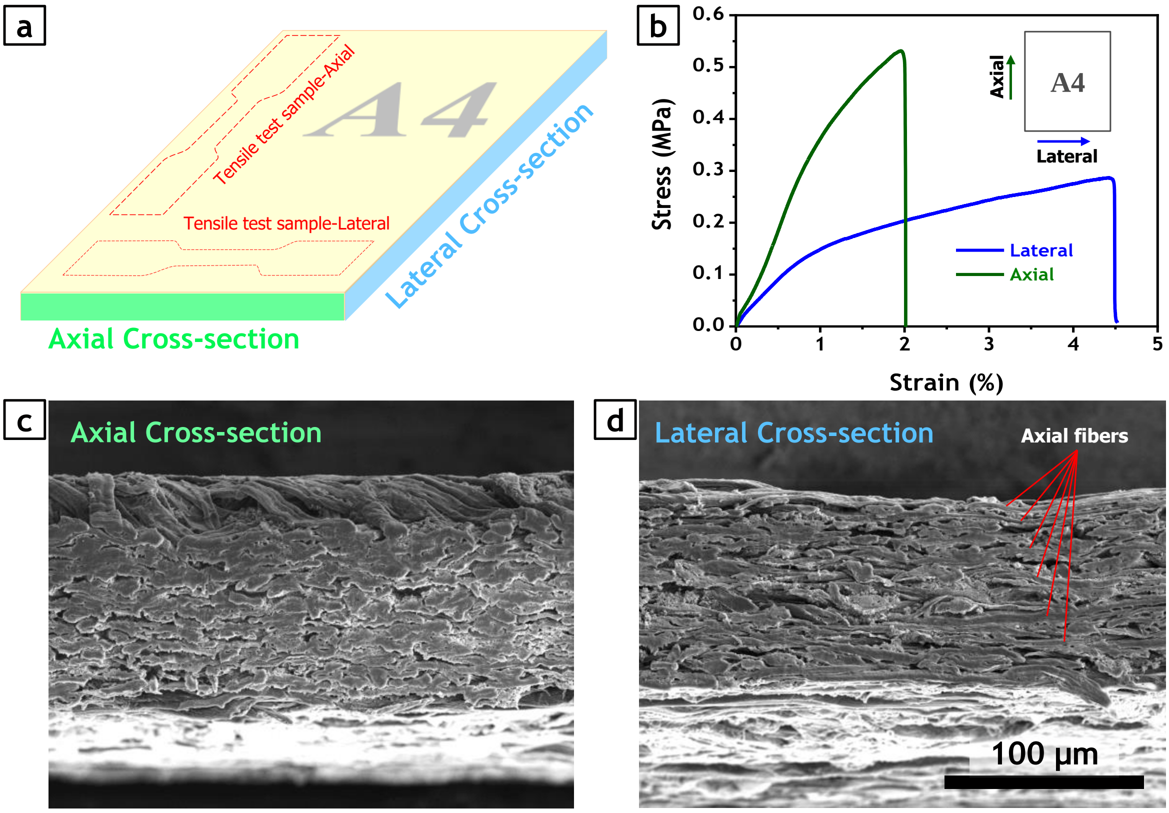

Figure S2. Characterizing orthotropic properties of commercial cellulose papers. a) Orientation of cutout samples for tensile test in axial and lateral directions in addition to schematic illustration of axial and lateral cross-sections. b) Stress-strain measurement for samples cut in axial and lateral directions. c) SEM image of axial cross-section of commercial cellulose paper. d) SEM image of lateral cross-section of commercial cellulose paper.

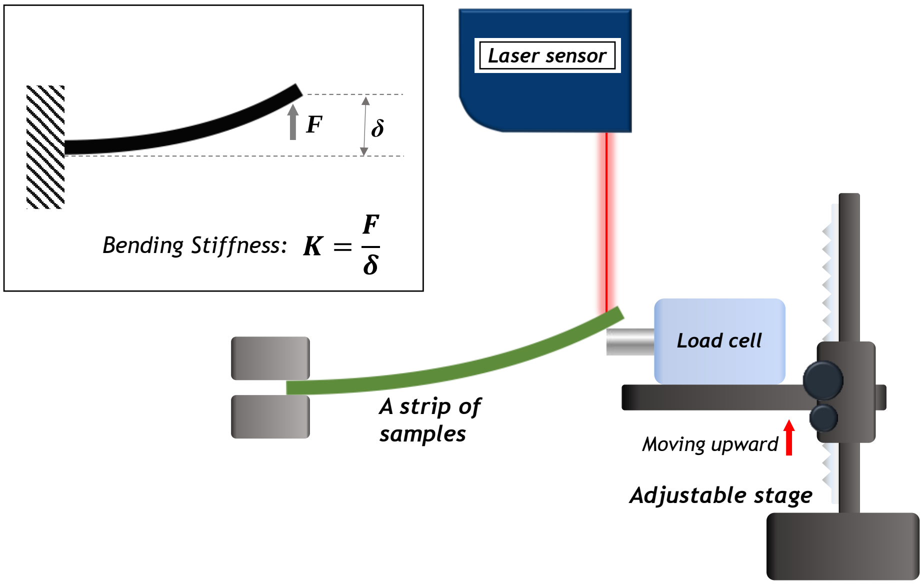

Figure S3. Measurement setup for calculating bending strain of samples.

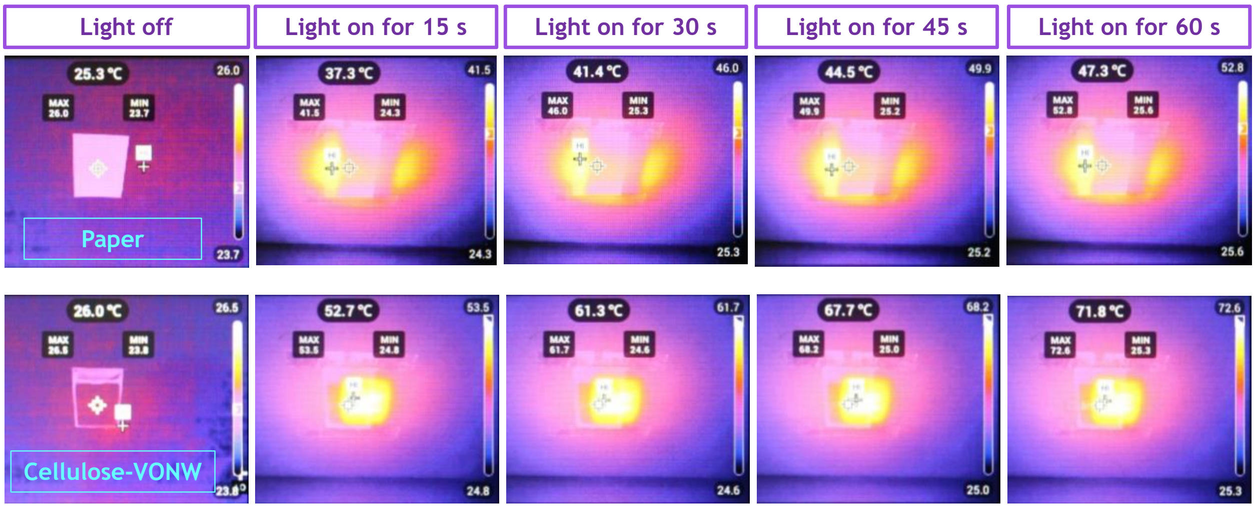

Figure S4. Thermal images of samples when exposed to light source at 10 cm distance.

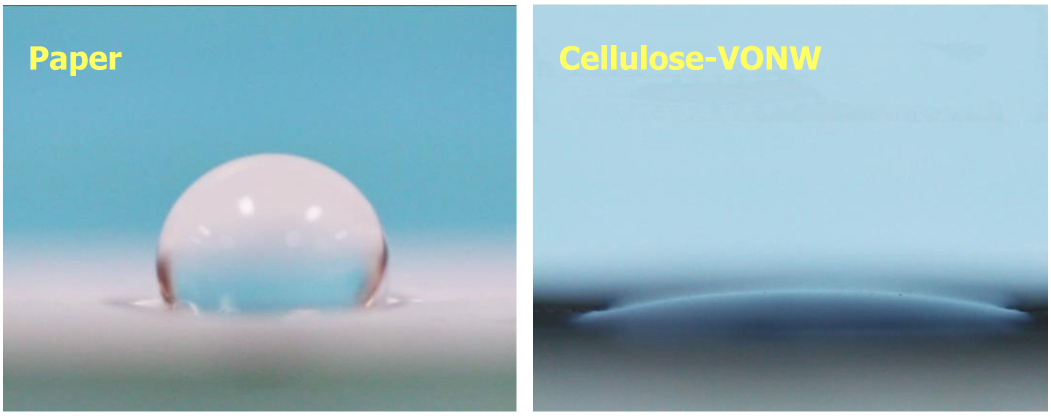

Figure S5. Contact angle measurement for water droplets on paper and on cellulose-VONW sample.

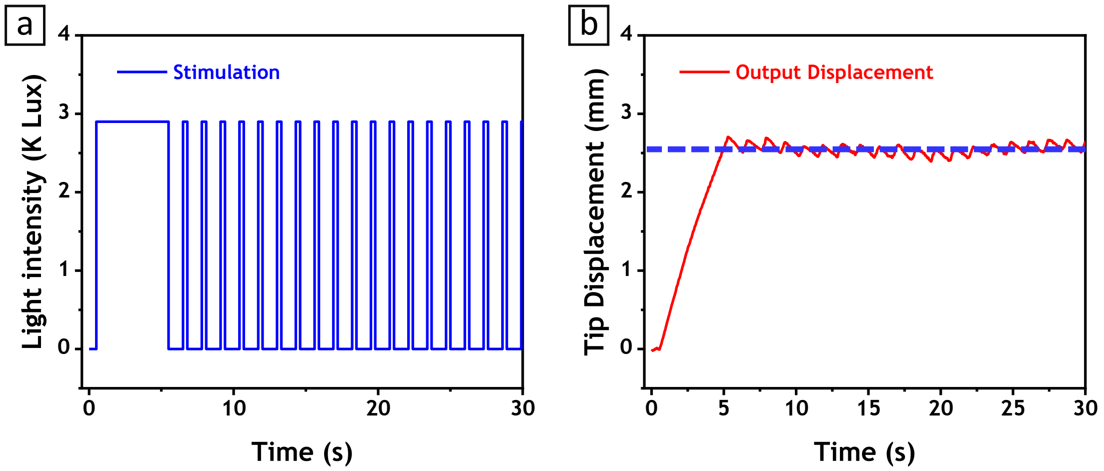

Figure S6. Adjusting the input stimulation for achieving constant displacement in any desirable level within the maximum limit of the actuator. a) Stimulation profile: constant stimulation for 5 s and then cyclic stimulation with the period of 1.3 s. b) measured output displacement in response to the designed stimulation.

Calculating Actuation Speed

To obtain the actuation speed, the first derivative of the displacement needs to be calculated. For this aim, the "*Forward Difference*" method can be utilized to differentiate the displacement data numerically:

$$V(t) = \frac{d\delta(t)}{dt} \tag{1}$$

$$\textit{Forward Difference} \quad \rightarrow \quad V(t) \approx \left|\frac{\delta(t+\Delta t)-\delta(t)}{\Delta t}\right| \tag{2}$$

where t denotes the time, $\delta(t)$ is the tip displacement of the actuator at moment t, and $V(t)$ represents the speed or velocity of the tip point at moment t. However, calculation of the speed is very sensitive to noise, such that the presence of slight noise in the signal leads to harsh fluctuations in the calculated speed. To avoid this issue, the noisy signal of the displacement is replaced by an estimated exponential function using Origin software. The estimated exponential function for the displacement of the actuator during cooling state is as follows:

$$\delta(t) = A_0 + A_1 e^{-t/b_1} + A_2 e^{-t/b_2} \tag{3}$$

As can be observed in **Figure S7**, the estimated function perfectly fits the displacement data and removes all noise. The calculated coefficients of the exponential function for the displacement of two samples, paper and cellulose-VONW, are presented in **Table S1**.

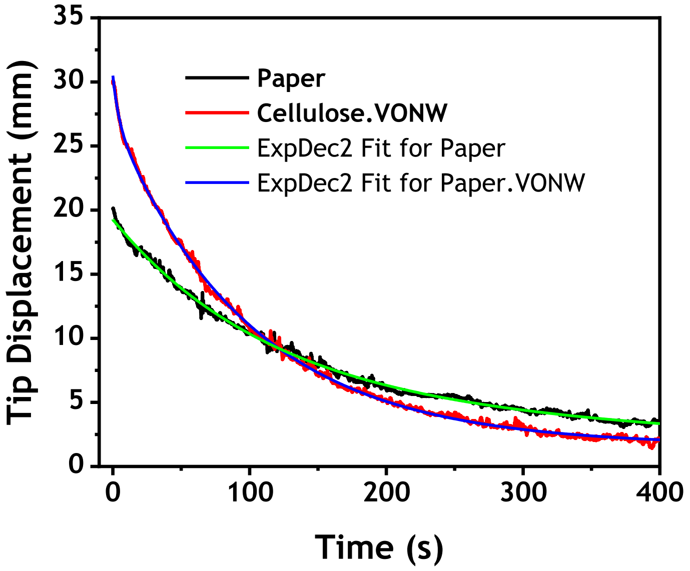

Figure S7. Estimated exponential functions for displacement of actuator during cooling state.

Table S1. Calculated coefficient of estimated exponential function for displacement.

Coefficients	cellulose-VONW	Paper
A_0	1.61653	-8352.05084
A_1	3.29446	14.76912
b_1	4.38876	116.52854
A_2	25.58231	8356.54724
b_2	99.93892	2050063.83277
R-Square	0.99902	0.99746

Table S2. Comparing the response time of proposed actuator with other actuators in the literature.

**Actuator**	**Response time**	**Recovery time**	**Reference**
Cellulose-VONW	60 s	300 s	This Work
Graphene-Based Polymer	30 s	No recovery	[1]
Polyvinyl alcohol microgel/ polyacrylic acid-coated biaxially oriented polypropylene	50 min	50 min	[2]
Silk–elastin-like protein hydrogel/ cellulose nanofibers	20 min	-	[3]
poly(N-isopropylacrylamide)/ poly(acrylic acid-co-acrylamide)	120 s	20 min	[4]
graphene oxide-poly(N-isopropylacrylamide)/ perylene bisimide-functionalized hyperbranched polyethylenimine	180 s	5 min	[5]
alginate-poly(2-(dimethylamino)ethyl methacrylate)	450 s	-	[6]
poly(N-isopropyl acrylamide)/ poly(acrylamide)	240 s	-	[7]
Graphene hydrogel	120 s	-	[8]
MoS_2	70 s	-	[9]
single-wall nanotube-PDMS	~65 s	-	[10]
GO-poly(N-isopropylacrylamide)/ poly(methylacrylic acid)	69 s	-	[11]

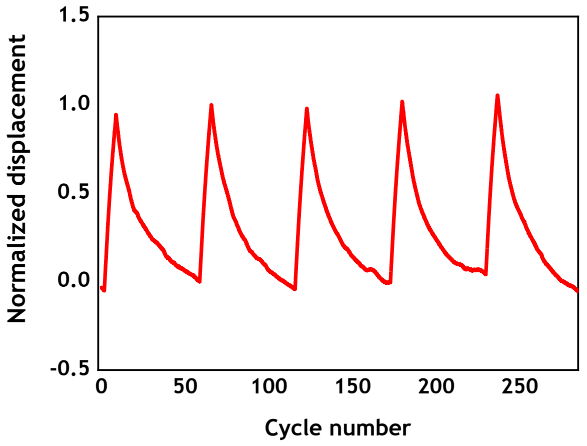

Figure S8. Response of cellulose-VONW actuator to cyclic stimulation

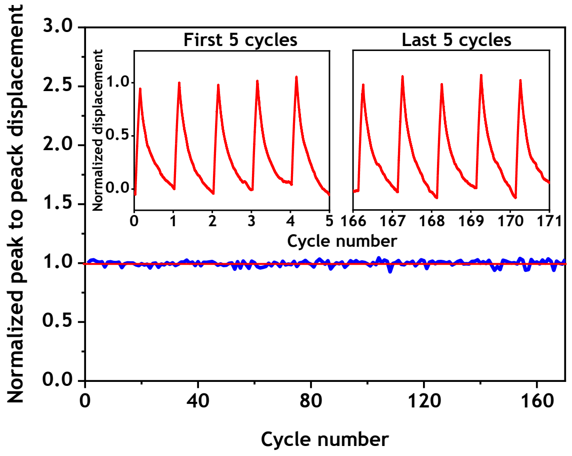

Figure S9. Cyclic stability of cellulose-VONW actuator during 170 cycles of stimulation

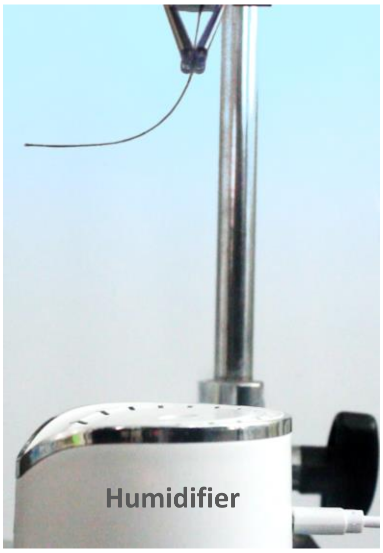

Figure S10. Experimental setup for vapor stimulation of the cellulose-VONW actuator

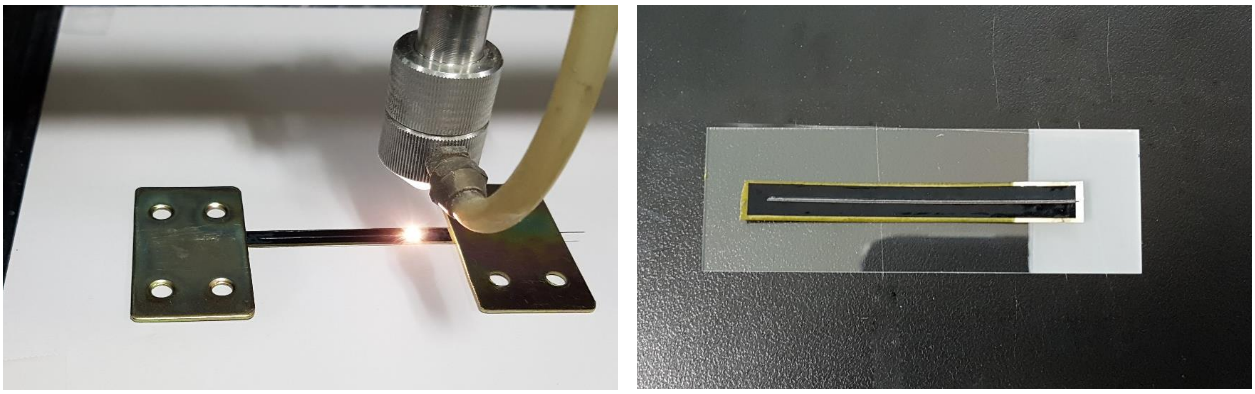

Figure S11. Engraving groove on PEDOT:PSS electrode using laser-cutter to make U-shape path for electron flow along surface of actuator.

Calculation of Average Electrical Power

As shown in **Figure S12**, by adding a constant resistor in series with the actuator, the electrical current can be calculated by measuring the voltage drop along the resistor:

$$I = I_r = \frac{V_r}{R}$$

In which I is the current in the whole circuit, I_r and V_r are the current and voltage of the resistor, respectively, and R is the electrical resistance of the resistor. Therefore, the exact voltage applied to the actuator can be calculated as follows:

$$V_a = V_{in} - V_r$$

where V_{in} and V_a are the input voltage and the voltage drop along the actuator, respectively. Now, the consumed electrical power can be calculated as:

$$P = I \times V_a$$

However, since the current is not constant during the time, the power value changes during actuation. Therefore, the average power is reported in Figure 5c during the 10 seconds of actuation.

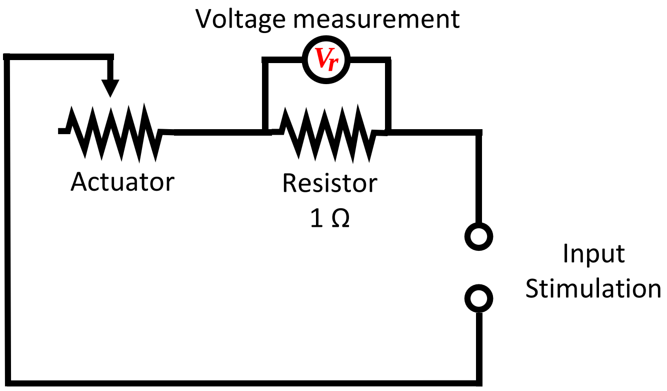

Figure S12. Electrical circuit for current measurement and consumed power calculations.

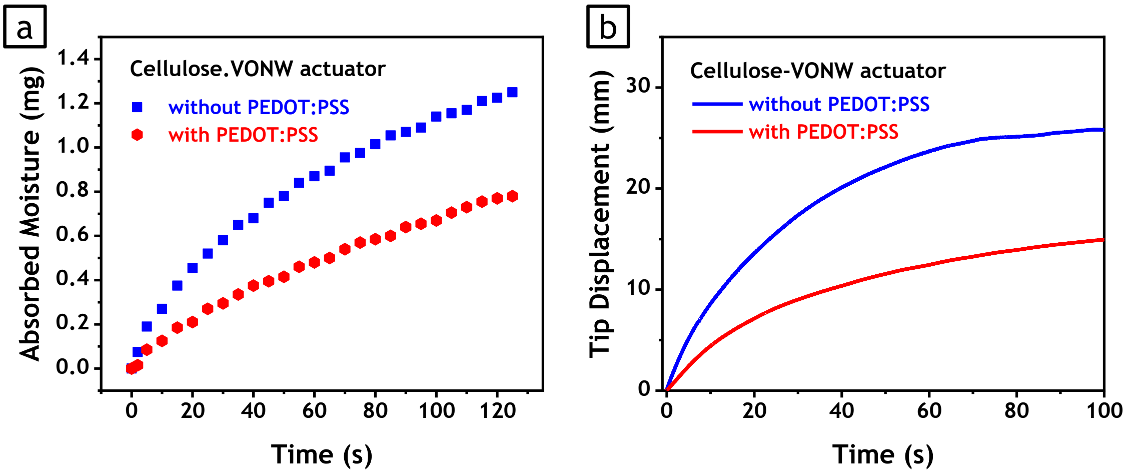

Figure S13. Effect of PEDOT:PSS coating on the water exchange and actuation of the actuator. a) Moisture absorption of cellulose-VONW actuator with and without PEDOT:PSS layer at room environment after being heated at 100° C for 1 hour. b) Response of cellulose-VONW actuator with and without PEDOT:PSS layer to the light stimulation in 10 cm distance.

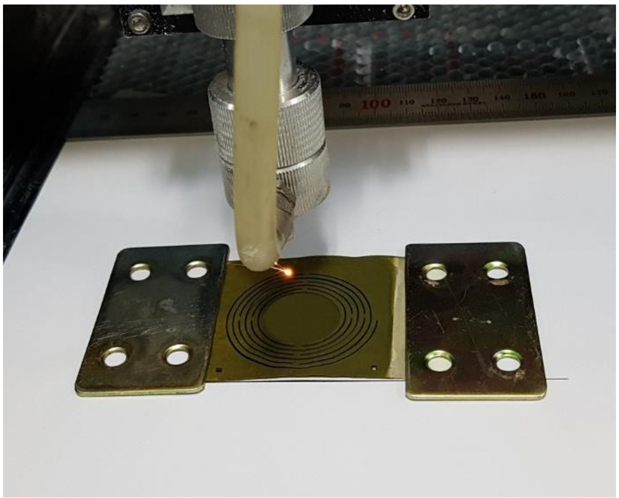

Figure S14. Cutting of kirigami pattern on cellulose-VONW film using laser-cutter.

References

[1] Tang, Zhenhua, et al. "Graphene-based polymer bilayers with superior light-driven properties for remote construction of 3D structures." *Advanced Science* 4.5 (2017): 1600437.

[2] Gao, Liang, et al. "Multi-responsive, bidirectional, and large deformation bending actuators based on borax cross-linked polyvinyl alcohol derivative hydrogel." *RSC advances* 7.63 (2017): 40005-40014.

[3] Wang, Yushu, et al. "Stimuli-responsive composite biopolymer actuators with selective spatial deformation behavior." *Proceedings of the National Academy of Sciences* 117.25 (2020): 14602-14608.

[4] Zheng, Jing, et al. "Mimosa inspired bilayer hydrogel actuator functioning in multi-environments." *Journal*

of Materials Chemistry C 6.6 (2018): 1320-1327.

[5] Ma, Chunxin, et al. "Bioinspired anisotropic hydrogel actuators with on–off switchable and color-tunable fluorescence behaviors." *Advanced Functional Materials* 28.7 (2018): 1704568.

[6] Zhou, Shengzhu, et al. "Ionic strength and thermal dual-responsive bilayer hollow spherical hydrogel actuator." *Macromolecular rapid communications* 41.8 (2020): 1900543.

[7] Shin, Yerin, et al. "Thermally triggered soft actuators based on a bilayer hydrogel synthesized by gamma ray irradiation." *Polymer* 212 (2021): 123163.

[8] Zhao, Ze, et al. "Bioinspired heterogeneous structural color stripes from capillaries." *Advanced Materials* 29.46 (2017): 1704569.

[9] Lei, Zhouyue, et al. "MoS_2 -based dual-responsive flexible anisotropic actuators." *Nanoscale* 8.44 (2016): 18800-18807.

[10] Lu, Shoaxin, et al. "Photo-mechanical actuation of carbon nanotubes: mechanisms and applications in micro and nano-devices." *Journal of Micro-Nano Mechatronics* 5.1 (2009): 29-41.

[11] Ma, Chunxin, et al. "A multiresponsive anisotropic hydrogel with macroscopic 3D complex deformations." *Advanced Functional Materials* 26.47 (2016): 8670-8676.
